# Book Reviews

**Published:** 1859-04

**Authors:** 


### BIOGRAPHICAL NOTICES.

### ARTICLE XI.

For the following sketches, we are greatly indebted to Dr. T. W. Evans, of Paris. They were sent to us some time since, but owing to accidental circumstances, have escaped our attention until recently.—Editors.

Le Maire.—Joseph Jean Francois Le Maire was born at Mayenne, 8th March, 1782; son of a celebrated surgeon in Brittany. He devoted himself when a child, to preserve, as an inheritance, the medical celebrity of his father, when the revolutionary troubles obliged him to seek refuge on board a government vessel. Being a member of a numerous family, he saw his hopes blasted at an age when one's life is generally full of bright illusions. A desire to contiuue his studies, (so sadly interrupted,) awakened for him the interest of the surgeon on board, who gave him some valuable counsel. When tranquillity wasrestored, he went to Paris and completed his medical studies at the college of medicine, where he became the fellow-student of Marjolin, Béclard, Cloquet, &c. He applied himself particularly to the profession of dentistry, and acquired in a short time a great reputation in this specialty.

Besides the progress he made in this science, the profession is indebted to him for a number of inventions and improvements in instruments in use in the dental department, which, at his epoch, where in a very imperfect state.

He died at Maisons, Alfort, 22d Feb., 1834. He was named Dentist to the King of Bavaria, Knight of the Legion of Honor, order of St. Hubert, &c.

His works are, viz. 1st. "The Ladies Dentist," containing advice from a man of merit to the mothers of families.

2d. "Treatise upon the Teeth, Physiologically." 3d. "Natural History of the Diseases of the Teeth," in two parts, with twenty-three wood cuts, by Joseph Fox. 4th. "Pathology," 2 vols. 5th. "Several Important Memoirs relative to the Art of Dentistry," published in the journals of that epoch.

M. DUVAL was born at Argantan, 1798. He pursued the study of medicine at Paris, and was made a member of the College of Surgery after having sustained a powerful thesis, under the presidency of the celebrated Professor Chopart, which was called "aniorismate varicose." Although doctor of medicine of the college at Paris, M. Duval applied himself more especially to the study of dentistry. He is also author of a number of works; a few of which we shall cite. He has made numerous researches which are of great importance, relative to diseases of the teeth. He divides them into three classes. 1st. Their tissue. 2d. Their connection. 3d. Their vital properties—first class is composed of the diseases of the hard parts, and those of the soft tissue. The diseases of the former are the cut, the fracture, the discoloration, the softening and swelling. Those of the soft parts are inflammation, suppuration, and ossification. The diseases of the connections comprise the loosening of the teeth, their mobility, their luxation. The swelling of the membrane, abcesses, &c. The diseases of the vital properties are the congealing by the cold air coming in contact, application of cold substances, their susceptibility to various impressions—rheumatism, neuralgia, &c. The observations of M. Duval upon the caries of the teeth, are divided into six series, and seven species, and are very instructive. The memoirs are entitled, viz. "Experience and Practical Observations upon Filled Teeth which are Susceptible to Galvanic Influence."—General Journal of Medicine, vol. 29, page 171. His "Researches and Observations upon the Dental Fistula." His work entitled "Accidents in Extracting Teeth," &c.—in 8vo. Paris, 1808. "Reflections upon Odontalgia."—8vo.,

Paris, 1808. "Historical Researches upon the Science of Dentistry among the Ancients."—Paris, 1808. Independent of all these works, M. Duval has published in most of the scientific journals, his curious researches upon the different affections of the teeth; in fact, all that this author has written upon the subject of dentistry is full of talent and interest.

Robert Bunon—a celebrated Dentist; born at Chalons, Sur Marne, May 1st, 1702; entered Saint Côrne in 1739, and died at Paris, January 25th, 1748. The three following works which he published have contributed greatly to the progress of his profession: "Dissertation upon the Pernicious Prejudices Relative to the Diseases of the Eyes which sometimes occur to Women with Child."—Paris, 1741. The author shows that there is no more danger attending the extraction of canine teeth, than in the extracting of any others; and that the nerves of these teeth have nothing to do with the eyes, as is vulgarly believed by many. He likewise proves that there is no danger whatever for women enciente to have teeth extracted which cause them pain. Essay upon Diseases of the Teeth, wherein he proposes to secure from infancy their conformation.—Paris, 1743. Bunon indicates the stages which follow nature in the development of the teeth from the first to the second dentition; and maintains that the first teeth are not destitute of roots, but that absorption destroys these as the others develop themselves. He has also established a distinction between decay and corrosion of the teeth. Experience and observations serve as evidences and proofs in the Essay upon Diseases of the Teeth.—Paris, 1746. A series of observations upon diseases of the teeth, particularly upon their corroding, were collected in the presence of a committee, appointed by the Academy of Surgeons.—(*Med. Biog.*)

Bourdet, *Dentist.*—He was received at college after having passed a great part of his youth following courses of surgery with skillful professors, and at the hospitals. Nevertheless he was not long in giving himself exclusively up to dentistry—drawn by an irresistible attraction towards this branch of surgery, in which he soon acquired a well merited reputation. He has left several works, among which we find "A Letter upon the Teeth—Paris, 1754," and a work very remarkable in 2 vols., published at Paris in 1757, under the title of "Researches and Observations upon every part of Dentistry." This work is full of judicious remarks of incontestible use. It contains also several wood cuts representing different instruments perfected or invented by the author. It is divided into seven chapters—each composed of different sections. The first under the name of "Physiology of the Teeth," containing the anatomy of the jaw, and the means to correct the malformation of the teeth. 2d. Treats of the various diseases which destroy the substance of the teeth—their internal and external causes—the means to prevent them—the remedies to be employed. In the 3d chapter, he discusses the diseases which change the whiteness of the teeth. The diseases of the alveoli, those of the gums, and their cure forms the 4th chapter. The 5th, includes the different operations which are practiced upon the teeth. The 6th, is the complete manual for the dentist, relative to artificial teeth, &c. And the 7th chapter, consists of some compositions of opiates, essences, and powders proper to preserve the teeth.

Louis Laforgue—a skillful Dentist, received at the college of surgery, at Paris; and dentist to the poor of the department of the Seine. Louis Laforgue is author of several works, of which the following are the titles: "Seventeen Articles Relative to the Diseases of the Teeth," in which he shows that many diseases are frequently attributed to the mouth, when the inspection of the mouth has proved that individual constitution is the source of the disease. "Theory put in Practice for the Treatment of the Teeth and Designation of the Diseases to which it is Applicable."—

Paris, 1800, in 8vo.; 204 pages. "Dissertation upon the Art of Preserving the Teeth."—1788 or 1790. "Theory and Practice of the Art of Dentistry," with 20 wood cuts—representing some instruments, teeth, sets of teeth and surgical instruments.—Paris, 1810 ; 2 vols., with a portrait of the author. "The Triumph of the First Dentition." "New and Curious Almanac for the Bissextile Year."—Paris, 1816 ; in 24. The writings of M. Laforgue contain some observations and precepts held by sound practice. They also contain a great number of assertions, paradoxical ; and the author has sometimes tainted them with censures, indifferent whether against his fellow practitioners or the physicians who had spoken of his works.

LEROY DE LA FAUDIGUIERE has published only a pamphlet, of 32 pages, upon the diseases of the gums and teeth.—Paris, 1775. A memoir for the college of surgery, relative to his elixir and opiate for the mouth—3 pages.

